# Douleur du coude chez un jeune sportif révélant un ostéome ostéoïde de l'apophyse coronoïde: à propos d'un cas

**DOI:** 10.11604/pamj.2015.22.45.7161

**Published:** 2015-09-17

**Authors:** Redouane Ouakrim, Younes Ouchrif, Issam El Ouakili, Mohammed Kharmaz, Moulay Omar Elamrani, Mohammed Elouadghiri, Mustapha Mahfoud, Ahmed Elbardouni, Abdou Lahlou, Mohammed Saleh Berrada

**Affiliations:** 1 Service de Chirurgie Orthopédique et Traumatologique, CHU de Rabat, Rabat, Maroc

**Keywords:** Apophyse coronoïde, ostéome ostéoïde, coude, coronoid process, osteoid osteoma, elbow

## Abstract

L'ostéome ostéoïde de l'apophyse coronoïde est exceptionnel, pose des problèmes diagnostiques et thérapeutiques. La douleur représente le maître symptôme. La perte d'extension complète est classique, cependant celle de la pronation et supination est très rare. Le scanner constitue l'examen radiologique de référence à condition de réaliser des coupes fines. La résection monobloc à foyer ouvert constitue le traitement de référence. Les traitements percutanés sont aussi efficaces mais au coude la proximité des éléments vasculo-nerveux et du cartilage articulaire rendent leurs indications plus limitées.

## Introduction

L'ostéome ostéoïde est une tumeur osseuse bénigne à histogenèse osseuse à l'origine d'une symptomatologie douloureuse caractéristique. Il se localise essentiellement au membre inférieur, en particulier au fémur et au tibia dans environ 80% des cas [1]. La localisation au coude est rare et celle de l'apophyse coronoïde est exceptionnelle, en effet seuls quelques cas isolés sont décrits dans la littérature [2], posant des problèmes diagnostiques et thérapeutiques. Nous rapportons le cas d'un exceptionnel ostéome ostéoïde de l'apophyse coronoïde chez un jeune basketteur de 28 ans, chez qui une résection monobloc de la tumeur a été réalisée.

## Patient et observation

Un jeune basketteur de 28 ans, droitier, a présenté depuis un an une douleur mixte du coude droit, sans irradiation particulière, partiellement soulagée par la prise d'anti-inflammatoires non-stéroïdiens et accompagnée d'une impotence fonctionnelle de plus en plus importante retentissant sur son activité sportive.Ces douleurs ont été longtemps rapportées à une épicondylite du coude. L'examen physique trouve une douleur à la palpation de la partie antéro-interne du coude, un déficit d'extension d'environ 20° par rapport au côté controlatéral, la flexion et la prono-supination étaient libres et complètes.

**Figure 1:**
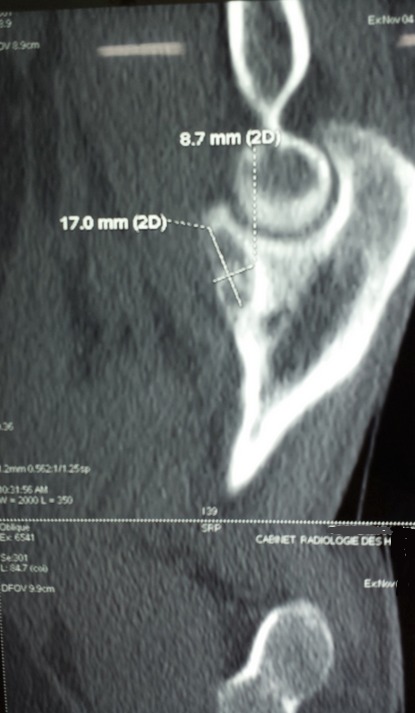
Aspect TDM de la tumeur: image typique en cocarde

**Figure 2:**
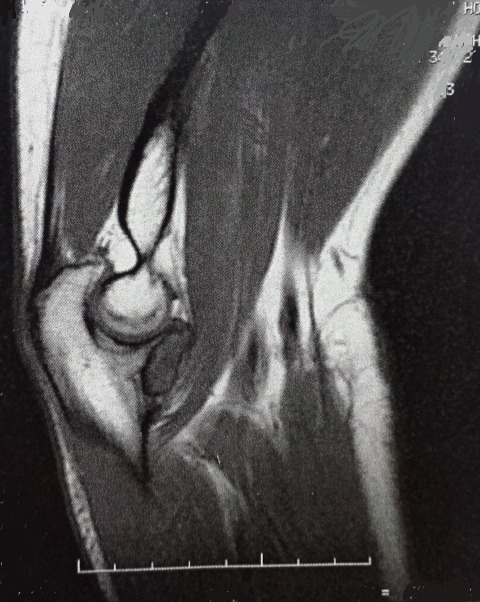
Aspect de la tumeur en hyposignal en T1 (IRM)

**Figure 3:**
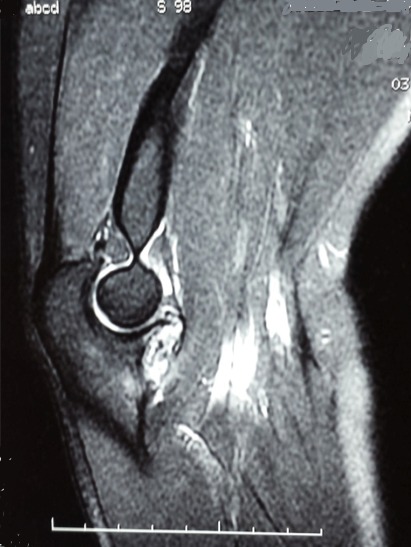
Aspect en hypersignal en T2 (IRM)

La radiographie standard du coude de face et de profil était sans particularités. Le scanner réalisé a montré une image lacunaire ovalaire entourant un foyer de calcification réalisant un aspect typique en cocarde, de siège cortical et affleurant le cartilage articulaire de la grande cavité sigmoïde de l'ulna (Figure 1), l'ostéosclérose réactionnelle était minime. En IRM, la lésion est en hypo signal en T1 et hyper signal en T2 (Figure 2 et Figure 3).

**Figure 4:**
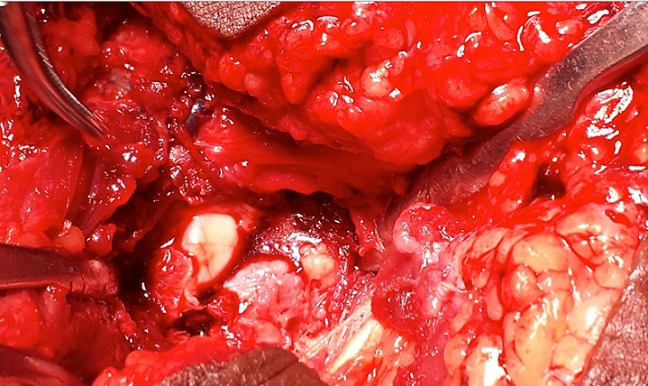
Aspect per opératoire de la tumeur

**Figure 5:**
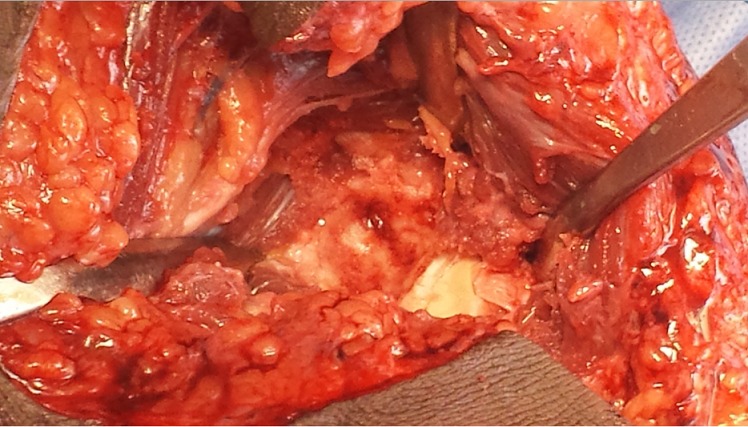
Aspect après résection de la tumeur

**Figure 6:**
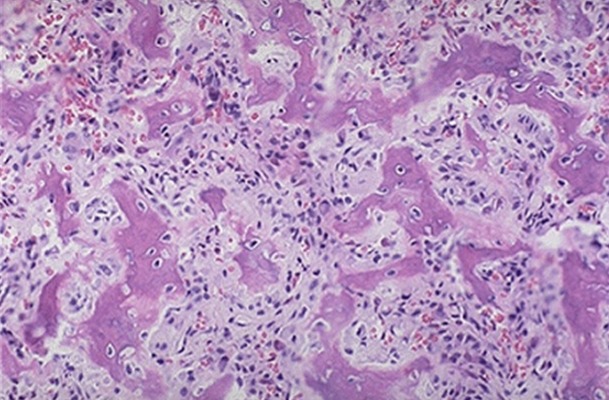
Étude anatomopathologique confirmant le diagnostic d'ostéome ostéoïde

Par une voie d'abord antéro-interne du coude et après dissection du pédicule huméral et du nerf médian qui sont écartés en dehors, l'apophyse coronoïde est visualisée en incisant dans le sens des fibres, le tendon du brachial antérieur. La tumeur avait un aspect bleuâtre affleurant le cartilage articulaire sans l'envahir (Figure 4). Le curetage complet de la lésion a laissé en place une cavité résiduelle comblée par un greffon osseux autologue (Figure 5). L'examen anatomopathologique a confirmé le diagnostic d'ostéome ostéoïde (Figure 6).

Les suites opératoires étaient simples. Après un recul de 8 mois la symptomatologie douloureuse a complétement disparu, le patient a pu reprendre son activité sportive progressivement à partir du 3^ème^ mois, mais il garde toujours un déficit d'extension d'environ 10 degrés.

## Discussion

L'ostéome ostéoïde décrit pour la première fois par Jeff [3] en 1935, est une tumeur osseuse bénigne caractérisée par une structure spécifique, le nidus hyper vascularisé entouré d'une ostéogénèse périphérique réactionnelle d'importance variable. Il représente 4% de l'ensemble des tumeurs osseuses primitives, survient de la seconde enfance à l’âge adulte avec une prédominance masculine soulignée dans toutes les séries [4].

La localisation au coude est rare. Becker et al [5] a colligé 33 cas d'ostéome ostéoïde du coude dans la littérature. L'atteinte de l'apophyse coronoïde est encore plus exceptionnelle, Masquelet et al [6] ont décrit le premier cas en 1986. La douleur est le maitre symptôme, typiquement d'horaire nocturne, soulagée par la prise d'aspirine. La caractéristique au coude est la présence d'une synovite réactionnelle aggravant cette douleur et pouvant entrainer une destruction cartilagineuse si le diagnostic est fait tardivement [7]. La perte de l'extension complète du coude est classique, par contre celle de la pronation et supination est exceptionnelle. Becker et al [5] ont décrit un cas avec perte de la pronation après résection de la lésion, Arne Decramer et al [4] rapportent le seul cas avec perte de la supination due à une atteinte de l'articulation radio cubitale supérieure.

La radiologie standard est une étape incontournable au diagnostic, montre typiquement une petite image claire entourée d'un halo de condensation. Le centre de l'image claire est parfois calcifié réalisant une image en cocarde. Le scanner constitue l'examen principal à condition de réaliser des coupes jointives fines [8]. A la scintigraphie, le nidus capte intensément les traceurs radioactifs, examen très sensible mais peu spécifique. L'IRM a peu d'intérêt dans le diagnostic de cette tumeur.

Le succès du traitement de cette tumeur dépend du repérage précis du nidus et de sa résection complète. Chez notre patient, vu le siège antérieure de la lésion et sa proximité du pédicule huméral et du nerf médian, nous avons opté pour un abord chirurgical antéro interne sans repérage préalable scannoguidé [8], ce qui a permis une résection complète de la tumeur, et une étude anatomopathologique permettant le diagnostic de certitude. Un geste de reconstruction au moyen d'un greffon osseux autologue était jugé nécessaire, vu la perte de substance osseuse engendrée par le curetage et pour prévenir une éventuelle instabilité du coude post- opératoire.

La résection percutanée décrite par KHOLER [8] nécessite un repérage TDM préalable du nidus, et expose à des complications telles que la nécrose cutanée, brûlures de la peau, atteinte neurologique, ostéomyélite et fractures [9]. La photo coagulation interstitielle au Laser, détruit la tumeur par chauffage. Il s'agit d'une technique inadaptée chez notre patient vu la proximité de la lésion au cartilage articulaire et aux éléments vasculo-nerveux. Martel et al [10] ont conclu que la présence d'une corticale osseuse intacte, protégeait les structures de voisinage, en particulier le cartilage articulaire durant la photo coagulation. Elle ne permet pas une étude anatomopathologique.

L’évolution est marquée par la possibilité de récidive, due le plus souvent à une résection incomplète du nidus. Toutefois, il a été décrit dans la littérature des cas de récidive après résection complète [11].

## Conclusion

L'ostéome ostéoïde de l'apophyse coronoïde est une lésion exceptionnelle, c'est un diagnostic auquel il faut penser devant une douleur inexpliquée du coude chez un jeune sportif.
